# Depleting long noncoding RNA HOTAIR attenuates chronic myelocytic leukemia progression by binding to DNA methyltransferase 1 and inhibiting PTEN gene promoter methylation

**DOI:** 10.1038/s41419-021-03637-4

**Published:** 2021-05-03

**Authors:** Haiping Song, Lijuan Chen, Wei Liu, Xiaoli Xu, Yongming Zhou, Jianhua Zhu, Xuexing Chen, Ziping Li, Hao Zhou

**Affiliations:** 1 Breast and Thyroid Surgery Center, Union Hospital, Tongji Medical College, Huazhong University of Science and Technology, Wuhan, China; 2 Department of Obstetrics and Gynecology, Union Hospital, Tongji Medical College, Huazhong University of Science and Technology, Wuhan, China; 3 Institute of Hematology, Union Hospital, Tongji Medical College, Huazhong University of Science and Technology, Wuhan, China; 4 Department of Hematology, Foshan No.1 People’s Hospital, Fosan, China; 5 Department of Hematology, The Affiliated Tianyou Hospital, Wuhan University of Science and Technology, Wuhan, China; 6 Laboratory of Clinical Immunology, Wuhan No. 1 Hospital, Tongji Medical College, Huazhong University of Science and Technology, Wuhan, China

**Keywords:** Cell biology, Diseases

## Abstract

Long noncoding RNAs (lncRNAs) are known to play a key role in chronic myelocytic leukemia (CML) development, and we aimed to identify the involvement of the lncRNA HOX antisense intergenic RNA (HOTAIR) in CML via binding to DNA methyltransferase 1 (DNMT1) to accelerate methylation of the phosphatase and tensin homolog (PTEN) gene promoter. Bone marrow samples from CML patients and normal bone marrow samples from healthy controls were collected. HOTAIR, DNMT1, DNMT3A, DNMT3B, and PTEN expression was detected. The biological characteristics of CML cells were detected. The relationship among HOTAIR, DNMT1, and PTEN was verified. Tumor volume and weight in mice injected with CML cells were tested. We found that HOTAIR and DNMT1 expression was increased and PTEN expression was decreased in CML. We also investigated whether downregulated HOTAIR or DNMT1 reduced proliferation, colony formation, invasion, and migration and increased the apoptosis rate of CML cells. Moreover, we tested whether low expression of HOTAIR or DNMT1 reduced the volume and weight of tumors in mice with CML. Collectively, the results of this studied showed that depleted HOTAIR demonstrated reduced binding to DNMT1 to suppress CML progression, which may be related to methylation of the PTEN promoter.

## Introduction

Chronic myeloid leukemia (CML) is a myeloproliferative neoplasia triggered by a translocation of reciprocal chromosomes 9 and 22, leading to the generation of a BCR/ABL fusion protein^1^. CML accounts for 20% of all adult leukemias and is diagnosed at a median age of about 50 years^2^. CML is featured by an initial chronic phase (CP) followed by the development to accelerated (AP) and blast crisis (BC) phase, the latter always resulting in patient death^3^. The diagnosis of CML needs the detection of the BCR/ABL oncoprotein, which is present in 95% of CML patients before the introduction of BCR/ABL as a diagnostic criterion^4^. Tyrosine kinase inhibitors are essential drugs for CML therapy, which could notably ameliorate the prognosis of CML patients^5^. However, most patients only enjoy a short response time, and drug resistance and clinical relapse develop fleetly in the advanced phases of CML post treatment, which are the main limitations for leukemia treatment^6^.

Long noncoding RNAs (lncRNAs) are an important series of non-coding RNAs with a length of 200 nucleotides^7^. LncRNA HOX transcript antisense intergenic RNA (HOTAIR) is transcribed from the homeobox (HOX) C locus, and it suppresses more distal HOXD sites and genes expression on other chromosomes, thus reducing the expression multiple genes, especially metastasis-inhibitor genes^8^. Epigenetic modulation of HOTAIR has been revealed in advanced CML^9^. Another study has demonstrated the role of HOTAIR in the acquired resistance to imatinib in CML cells^10^. DNA methylation is the most studied epigenetic modification pattern which is mediated and maintained by DNA methyltransferases (DNMTs), while DNMT1 is necessary for methylation maintenance^11^. It is revealed that DNMT1 and DNMT3A expression are enhanced in cell lines and patients with advanced phase CML^12^. Another study has demonstrated that DNMT1 mediates abnormal methylation and downregulation of SHP-1 gene in CML cells^13^. As a protein phosphatase and bifunctional lipid, phosphatase and tensin homolog (PTEN) has been reported to attenuate cell growth, phagocytosis, and cytoskeletal remodeling^14^. According to Chen et al., the expression reconstitution of wild-type PTEN dramatically suppresses the invasion, migration, and proliferation abilities of CML K562 cells^15^. Moreover, it is presented that PTEN plays a crucial role in the pathogenesis of CML^16^. In this study, we aim to identify the involvement of HOTAIR in CML by binding to DNMTs to accelerate the methylation of PTEN promoter.

## Results

### HOTAIR and DNMT1 expression are raised, and PTEN expression is reduced in bone marrow of CML patients, and overexpression of HOTAIR is related to poor prognosis of CML patients

**Fig. 1:**
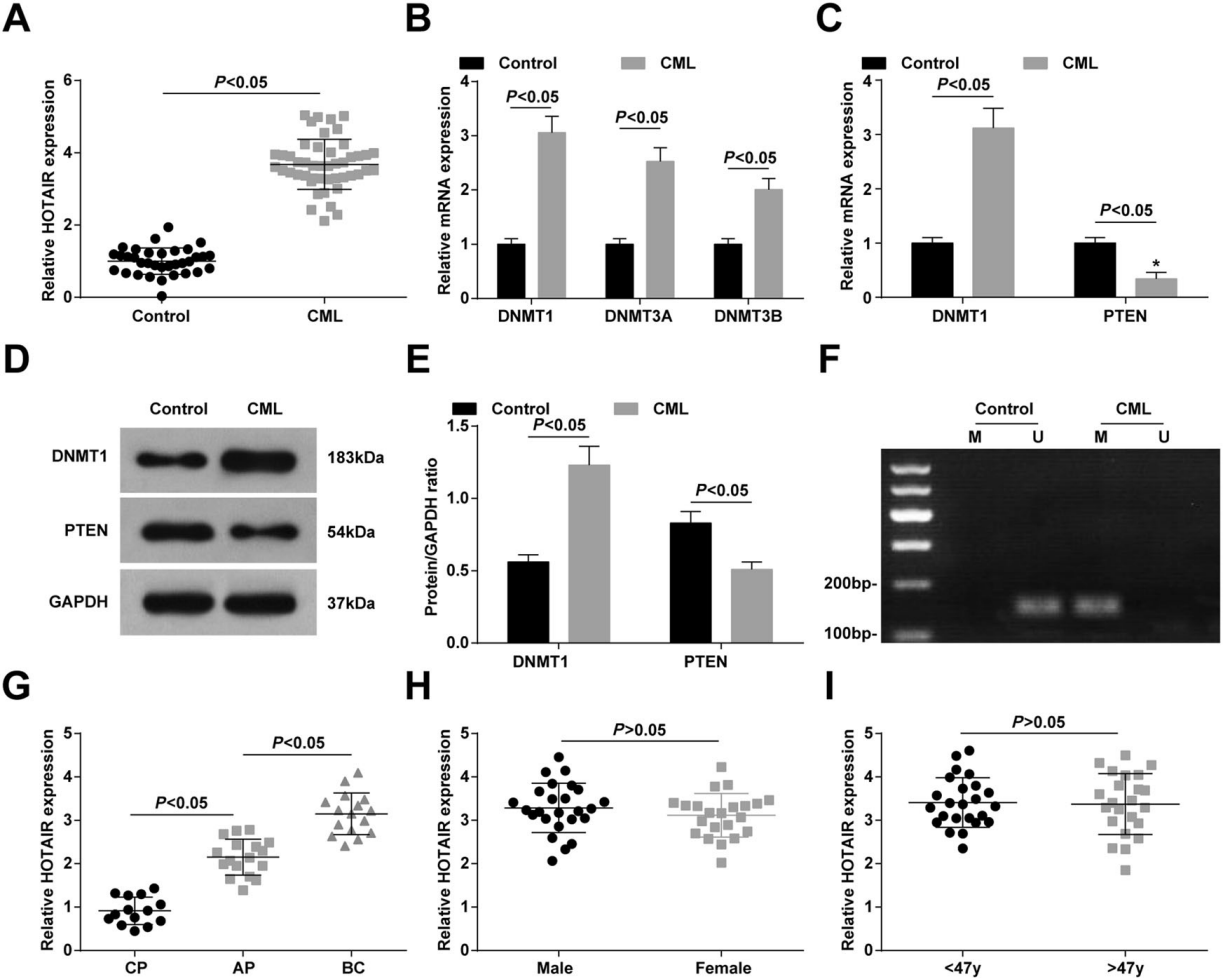
HOTAIR and DNMT1 expression are raised, and PTEN is reduced in bone marrow of CML patients, and overexpression of HOTAIR is related to poor prognosis of CML patients. **A** Expression of HOTAIR in patients with CML. **B** Expression of methyltransferase in patients with CML. **C** Expression of DNMT1 and PTEN mRNA in patients with CML. **D** Protein bands of DNMT1 and PTEN in patients with CML. **E** DNMT1 and PTEN protein expression levels in CML patients. **F** PTEN methylation in CML patients. **G** Effect of WHO classification on HOTAIR expression. **H** Effect of gender on HOTAIR expression. **I** Effect of age on HOTAIR expression (control group, *n* = 34; CML group, *n* = 47; CP group, *n* = 14; Ap group, *n* = 17; BC group, *n* = 16; male group, *n* = 25; Female group, *n* = 22; <47 y group, *n* = 24; >47 y group, *n* = 23). Measurement data were depicted as mean ± standard deviation, comparisons between two groups were conducted by independent sample *t*-test.

RT-qPCR, MSP, and western blot assay displayed that in bone marrow of CML patients, DNMT1, DNMT3A, and DNMT3B were heightened, among which the change of DNMT1 expression was the greatest, so DNMT1 was selected for follow-up experiments. HOTAIR and DNMT1 levels were enhanced, PTEN expression was reduced and PTEN methylation was enhanced in bone marrow of CML patients (all *P* < 0.05) (Fig. 1A–F).

CML patients were grouped according to WHO classification. RT-qPCR was utilized to detect HOTAIR expression in bone marrow of CML patients, revealing that HOTAIR expression was higher in BC phase than in CP phase (Fig. 1G). However, HOTAIR expression was not changed with age and gender (Fig. 1H, I).

### HOTAIR and DNMT1 expression are elevated, and PTEN expression is reduced in CML cells

**Fig. 2:**
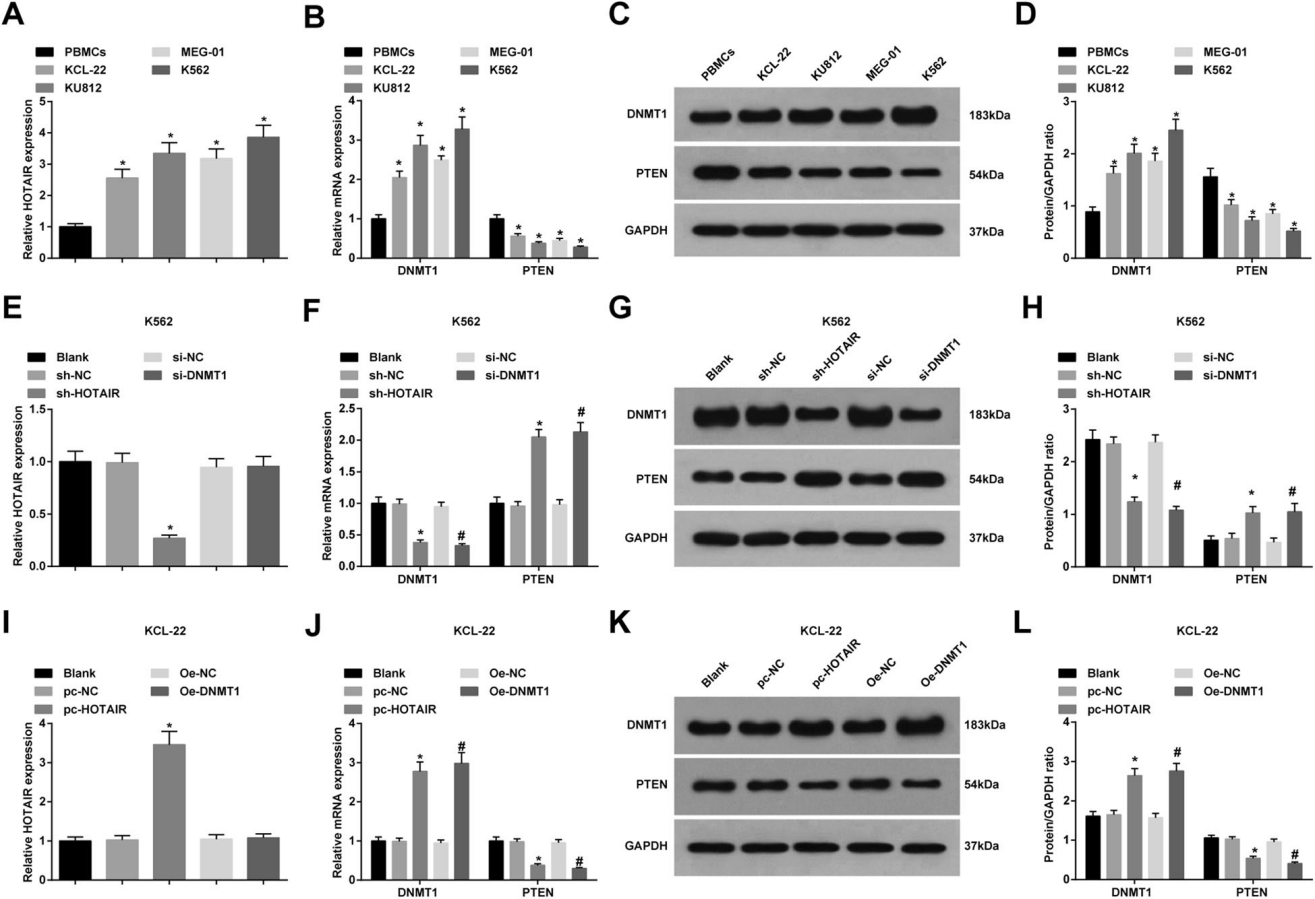
HOTAIR and DNMT1 expression are elevated, and PTEN is reduced in CML cells. **A** HOTAIR expression in CML cell lines in each group. **B** DNMT1 and PTEN mRNA expressions in CML cell lines in each group. **C** Protein bands of DNMT1 and PTEN in CML cell lines. **D** DNMT1 and PTEN protein expression in CML cell lines in each group. **E** HOTAIR expression in each group of K562 cells. **F** DNMT1 and PTEN mRNA expressions in each group of K562 cells. **G** Protein bands of DNMT1 and PTEN in each group of K562 cells. **H** DNMT1 and PTEN protein expression in each group of K562 cells. **I** HOTAIR expression in each group of KCL-22 cells. **J** DNMT1 and PTEN mRNA expressions in each group of KCL-22 cells. **K** Protein bands of DNMT1 and PTEN in each group of KCL-22 cells. **L** DNMT1 and PTEN protein expression in each group of KCL-22 cells. In CML cell lines, *P < 0.05 vs. the BMCs. In K562 cells, *P < 0.05 vs. the sh-NC group, ^#^*P* < 0.05 vs. the si-NC group. In KCL-22 cells, *P < 0.05 vs. the pc-NC group, ^#^*P* < 0.05 vs. the OE-NC group. *N* = 3. Measurement data were depicted as mean ± standard deviation. Comparisons among multiple groups were assessed by one-way ANOVA followed by Tukey’s multiple comparisons test.

In CML cell lines, in contrast with BMCs, HOTAIR and DNMT1 expression were enhanced, and PTEN expression was decreased in CML cells (all *P* < 0.05), among which K562 cells showed the greatest difference while KCL-22 cells showed the minimum difference with BMCs (Fig. 2A–D).

In K562 cells, versus the sh-NC group, HOTAIR and DNMT1 expression were decreased, and PTEN expression was raised in the sh-HOTAIR group (all *P* < 0.05). In comparison to the si-NC group, DNMT1 expression was reduced as well as PTEN expression was enhanced in the si-DNMT1 group (both *P* < 0.05) (Fig. 2E–H).

In KCL-22 cells, compared to the pc-NC group, HOTAIR and DNMT1 expression were elevated, and PTEN expression was degraded in the pc-HOTAIR group (all *P* < 0.05). Versus the OE-NC group, DNMT1 expression was raised, and PTEN expression was reduced in the OE-DNMT1 group (both *P* < 0.05) (Fig. 2I–L).

### Downregulated HOTAIR or downregulated DNMT1 reduces the proliferation, colony formation and cell cycle progression, as well as increases apoptosis rate of CML cells

**Fig. 3:**
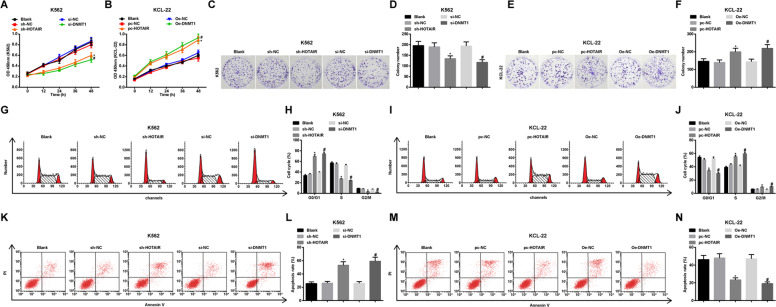
Downregulated HOTAIR or DNMT1 reduces the proliferation, colony formation and cell cycle progression, as well as increases apoptosis rate of CML cells. **A** K562 cell proliferation activity in each group. **B** KCL-22 cell proliferation activity in each group. **C** Colony formation of K562 cells in each group. **D** Number of colonies of K562 cells in each group. **E** Colony formation of KCL-22 cells in each group. **F** Number of colonies of KCL-22 cells in each group. **G** Cell cycle of K562 cells in each group. **H** K562 cell cycle ratio in each group. **I** Cell cycle of KCL-22 cells in each group. **J** KCL-22 cell cycle ratio in each group. **K** Apoptosis of K562 cells in each group. **L** Apoptosis rate of K562 cells in each group. **M** Apoptosis of KCL-22 cells in each group. **N** Apoptosis rate of KCL-22 cells in each group. In K562 cells, *P < 0.05 vs. the sh-NC group, ^#^*P* < 0.05 vs. the si-NC group. In KCL-22 cells, *P < 0.05 vs. the pc-NC group, ^#^*P* < 0.05 vs. the OE-NC group. *N* = 3. Measurement data were depicted as mean ± standard deviation. Comparisons among multiple groups were assessed by one-way ANOVA followed by Tukey’s multiple comparisons test.

CCK-8, colony formation assays and flow cytometry reported that: in K562 cells, in contrast to the sh-NC group and the si-NC group, respectively, cell proliferation, the number of colonies, S and G2/M phase cells were reduced while G0/G1 phase cells and apoptosis rate were remarkably enhanced in the sh-HOTAIR group and the si-DNMT1 group (all *P* < 0.05) (Fig. 3A, C, D, G, H, K, L). In KCL-22 cells, versus the pc-NC group and OE-NC group, respectively, cell proliferation, the number of colonies, S and G2/M phase cells were raised while G0/G1 phase cells and apoptosis rate were reduced in the pc-HOTAIR group and OE-DNMT1 group (all *P* < 0.05) (Fig. 3B, E, F, I, J, M, N).

### Depleted HOTAIR or DNMT1 attenuates invasion and migration of CML cells

**Fig. 4:**
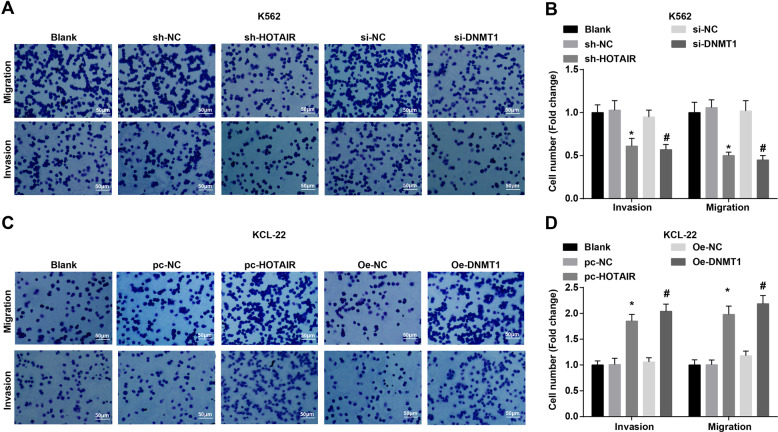
Depleted HOTAIR or DNMT1 attenuates invasion and migration of CML cells. **A** Invasion and migration of K562 cells in each group. **B** Invasion and migration of K562 cells in each group. **C** Invasion and migration of KCL-22 cells in each group. **D** Invasion and migration of KCL-22 cells in each group. In K562 cells, *P < 0.05 vs. the sh-NC group, ^#^*P* < 0.05 vs. the si-NC group. In KCL-22 cells, *P < 0.05 vs. the pc-NC group, ^#^*P* < 0.05 vs. the OE-NC group. *N* = 3. Measurement data were depicted as mean ± standard deviation. Comparisons among multiple groups were assessed by one-way ANOVA followed by Tukey’s multiple comparisons test.

The invasion and migration ability of CML cells were tested by Transwell assay, and the results demonstrated that in K562 cells, the number of invasive and migratory cells was decreased in the sh-HOTAIR group and the si-DNMT1 group relative to that in the sh-NC group and the si-NC group (all *P* < 0.05) (Fig. 4A, B). In KCL-22 cells, by comparison with the pc-NC group and OE-NC group, the number of invasive and migratory cells was enhanced in the pc-HOTAIR group and OE-DNMT1 group (all *P* < 0.05) (Fig. 4C, D).

### HOTAIR binds to DNMT1 to regulate promoter methylation of PTEN

**Fig. 5:**
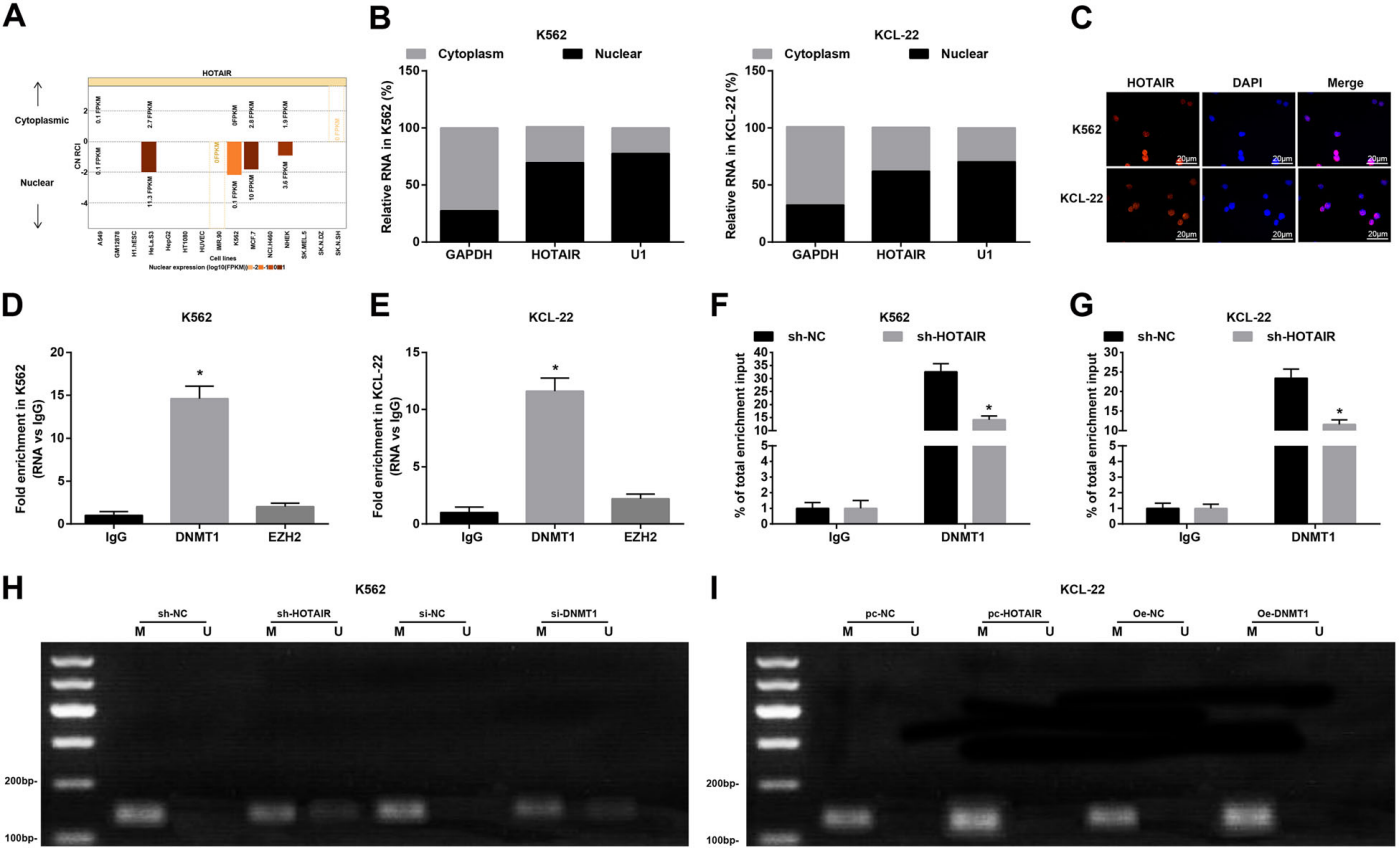
HOTAIR binds to DNMT1 to regulate PTEN methylation. **A** HOTAIR subcellular localization predicted by online analysis site. **B** HOTAIR distribution in K562 cells and KCL-22 c ells. **C** HOTAIR distribution in K562 cells and KCL-22 cells verified by FISH assay. **D** HOTAIR binds to methyltransferase in K562 cells tested by RIP assay. **E** HOTAIR binds to methyltransferase in KCL-22 cells tested by RIP assay. **F** The binding of DNMT1 and PTEN in K562 cells tested by ChIP assay. **G** The binding of DNMT1 and PTEN in KCL-22 cells tested by ChIP assay. **H** PTEN methylation level in K562 ce lls tested by MSP. **I** PTEN methylation level in KCL-22 cells tested by MSP. In RIP assay, *P < 0.05 vs. the IgG group. In ChIP assay, *P < 0.05 vs. the sh-NC group. *N* = 3. Measurement data were depicted as mean ± standard deviation. Comparisons between two groups were conducted by independent sample *t*-test, and comparison among multiple groups were assessed by one-way ANOVA followed by Tukey’s multiple comparisons test.

The results revealed by online analysis site (http://lncatlas.crg.eu/) and subcellular localization experiment showed that HOTAIR was mainly distributed in the nucleus of K562 and KCL-22 cells (Fig. 5A, B), which was verified by FISH assay (Fig. 5C). RIP assay presented that (Fig. 5D, E) HOTAIR mainly interacted with methyltransferase DNMT1 in K562 cells and KCL-22 cells. ChIP assay revealed that (Fig. 5F, G) DNMT1 was mainly bound to PTEN promoter in K562 cells and KCL-22 cells. In addition, MSP detection revealed that in K562 cells, versus with the sh-NC and si-NC groups, PTEN methylation level in the sh-HOTAIR and si-DNMT1 groups was reduced (both *P* < 0.05) (Fig. 5H). In KCL-22 cells, with respect to the pc-NC group and OE-NC group, PTEN methylation of pc-HOTAIR group and OE-DNMT1 group was increased (both *P* < 0.05) (Fig. 5I).

### Low expression of HOTAIR or DNMT1 reduces the volume and weight of tumor in mice injected with CML cells

**Fig. 6:**
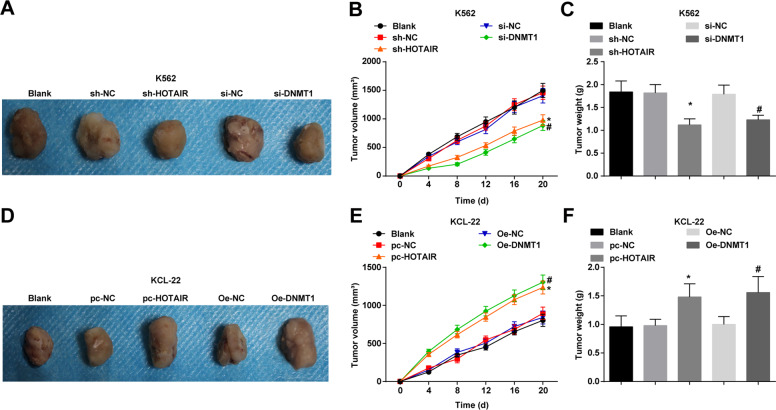
Low expression of HOTAIR or DNMT1 reduces the volume and weight of tumor in mice injected with CML cells. **A** K562 cell xenografts in each group. **B** Tumor volume change of K562 cell xenografts in each group. **C** The tumor weight of K562 cell xenografts in each group. **D** KCL-22 cell xenografts in each group. **E** Tumor volume change of KCL-22 cell xenografts in each group. F, The tumor weight of KCL-22 cell xenografts in each group. In K562 cells, *P < 0.05 vs. the sh-NC group, ^#^*P* < 0.05 vs. the si-NC group. In KCL-22 cells, *P < 0.05 vs. the pc-NC group, ^#^*P* < 0.05 vs. the OE-NC group. *n* = 3. Measurement data were depicted as mean ± standard deviation. Comparisons among multiple groups were assessed by one-way ANOVA followed by Tukey’s multiple comparisons test.

Results of in vivo assay indicated that in K562 cells, versus the sh-NC group and the si-NC group, the tumor volume and weight were reduced in the sh-HOTAIR group and the si-DNMT1 group (all *P* < 0.05) (Fig. 6A–C). In KCL-22 cells, versus the pc-NC group and OE-NC group, the tumor volume and weight were raised in the pc-HOTAIR group and OE-DNMT1 group (all *P* < 0.05) (Fig. 6D–F).

## Discussion

CML is a myeloproliferative disease featured by the BCR-ABL fusion gene, which forms a chimeric protein with deregulated tyrosine kinase activity^17^. Also, a recent study has provided a proof that depletion of HOTAIR may play an essential role in ameliorating acquired resistance to imatinib in CML^10^. It is reported that DNMT1 drives transcriptional downregulation of β catenin antagonist Chibby1 linked to the BCR-ABL1 gene of CML^18^. In a study conducted by Panuzzo et al., it is shown that tumor inhibitor PTEN serves a key role in the pathogenesis of chronic phase CML by non genomic loss of function mechanisms^19^. Our aim is to investigate the involvement of HOTAIR in CML by binding to DNMTs to promote the methylation of PTEN gene promoter.

In our study, HOTAIR, DNMT1, and PTEN as well as PTEN methylation were detected and the results reported that HOTAIR and DNMT1 expression were raised, while PTEN was reduced in CML cells and bone marrow of CML patients. Meanwhile, upregulated HOTAIR was related to poor prognosis of CML patients. A recent study has presented that HOTAIR expression was markedly elevated in all CML patients relative to that in health donors^10^. Another study has presented that the advanced stage of CML was linked to the high levels of HOTAIR, DNMT1, and DNMT3A^9^. It is presented that DNMT1 expression was dramatically raised in patients in CML-AP and CML-BP versus the controls^20^. It is reported that the expression of PTEN was reduced, whereas DNMT1 was raised in the AP and BP of CML^21^. Furthermore, it is revealed that PTEN protein expression was notably reduced in CML patients in AP/BP^22^. Our study also suggested that HOTAIR bound to DNMT1 in CML cells. A study has reported that HOTAIR and DNMT1 were both up-regulated in CML cells^9^, but did not reveal the binding relationship between HOTAIR and DNMT1. Another studies has reported that downregulating HOTAIR degraded DNMT1 protein expression in prostate cancer cells and osteosarcoma cells^23,24^. However, the binding relationship between HOTAIR and DNMT1 in CML needs further study.

Additionally, our data reported that depleted HOTAIR or DNMT1 reduced the proliferation, colony formation, invasion, migration, as well as increased apoptosis rate of CML cells, while low expression of HOTAIR or decreased DNMT1 reduced the volume and weight of tumor in mice injected with CML cells. It has been previously suggested that downregulated HOTAIR attenuated proliferation and enhanced apoptosis in KCL22 and K562 cells^9^. It is reported that depleting HOTAIR in acute myeloid leukemia (AML) reduced cell proliferation and induced apoptosis in vivo and in vitro^25^. It is displayed that low expression of HOTAIR suppressed cell growth and the number of colony formation as well as promoted apoptosis in AML^26^. Another study also demonstrated that silencing HOTAIR inhibited cell proliferation in leukemia cells^27^. Also, it has been verified that in K562 cells, DNMT1 downregulation led to cell growth inhibition and induction of caspase‑3‑dependent apoptosis^28^. A prior research has confirmed that inhibition of DNMT1 triggered growth inhibition and apoptosis in multiple myeloma cell lines^29^. Moreover, downregulation of DNMT1 in human tumor xenografts reduced in vivo tumor growth^30^. All these evidence suggests the combined effect of HOTAIR and DNMT1 in CML development.

To briefly conclude, our study provides evidence that depleted HOTAIR could inhibit its binding to DNMT1, thereby suppressing the proliferation and facilitating apoptosis of CML cells, which is related to regulation of methylation of PTEN promoter. These findings provide a new insight into a novel target therapy for CML. Nevertheless, further efforts are still needed.

## Materials and methods

### Study subjects

From January 2014 to December 2014, bone marrow samples were collected from CML inpatients of Union Hospital, Tongji Medical College, Huazhong University of Science and Technology. All cases were diagnosed by clinical examination, cell morphology analysis, immunology, and histochemical staining. Forty-seven patients had CML, including 25 males and 22 females, with an average age of 47.5 (16.7–78.6) years. The diagnostic type of CML was in accordance with the World Health Organization (WHO) classification criteria^31^. Patients were divided into the CP (14 cases), AP (17 cases), and BC (16 cases) phases. All bone marrow samples were taken from the posterior superior iliac spine. Thirty-four bone marrow samples from the control group were taken from the posterior superior iliac spine of healthy volunteers (18 males and 16 females) with an average age of 38.7 (27.9–72.2) years. Heparin anticoagulant bone marrow (2 mL/sample) was collected, and bone marrow mononuclear cells (BMCs) were amassed and stored at −80 °C.

### Cell culture and screening

More than 90% of CML patients have Phl chromosomes in their blood cells, and the C-abl proto-oncogene on the long arm of chromosome 9 is translocated to chromosome 22 to form a BCR/ABL fusion gene^32–34^. KCL-22 cells were established from the pleural effusion of a 32-year-old woman with Philadelphia chromosome-positive CML in blast crisis and contained *t*(9;22), leading to the BCR-ABL1 e13-a2 (b2-a2) fusion gene and a p53 mutation. K562 cells established from the pleural effusion of a 53-year-old woman with CML in blast crisis carried the BCR-ABL1 e14-a2 (b3-a2) fusion gene; KU812 cells were established from the peripheral blood of a 38-year-old man with CML in myeloid blast crisis and expressed basophilic features and carried *t* (9;22), leading to the BCR-ABL1 e14-a2 (b3-a2) fusion gene; MEG-01 cells were established from the bone marrow of a 55-year-old man with CML in megakaryocytic blast crisis and carried *t* (9;22), leading to the BCR-ABL1 e13-a2 (b2-a2) fusion gene. Human CML cell lines (KCL-22, K562, KU812, and MEG-01) containing the BCR/ABL fusion gene were obtained from DSMZ (Braunschweig, Germany). BMCs were used as the control group. All the CML cell lines were cultured in Roswell Park Memorial Institute 1640 medium with 10 mL/dL fetal bovine serum (FBS) and 1 g/dL penicillin-streptomycin. The medium was changed every 2–3 days. K562 cells with the greatest expression difference compared to BMCs and KCL-22 cells with the minimum expression difference compared to BMCs were selected for subsequent experiments.

### Cell grouping and transfection

K562 and KCL-22 cells were seeded in a 6-well plate at 3 × 10^5^ cells/well. Cells were transfected when they reached approximately 90% confluence. Transfection was performed with Lipofectamine 2000 reagent according to the manufacturer’s instructions (Invitrogen, CA, USA): 3 mL serum-free medium was utilized to dilute each transfection reaction (all from RiboBio, Guangdong, China, and the final concentration was 50 nM). Another 3 mL serum-free medium was utilized to dilute 600 µL Lipofectamine 2000. The above two mixtures were mixed and added to cells for 6 h. Then, the medium was changed to the corresponding culture medium containing 10% FBS for subsequent experiments.

K562 cells were assigned into five groups: the blank group (no treatment), short hairpin RNA (sh)-HOTAIR group (transfection of the sh-HOTAIR vector), sh-negative control (NC) group (transfection of the sh-HOTAIR vector NC), small interfering RNA (si)-DNMT1 group (transfection of the si-DNMT1 vector), and si-NC group (transfection of the si-DNMT1 vector NC).

KCL-22 cells were divided into five groups: the blank group (no treatment), pc-HOTAIR group (transfection of the pc-HOTAIR vector), pc-NC group (transfection of the pc-HOTAIR vector NC), overexpression (OE)-DNMT1 group (transfection of the OE-DNMT1 vector), and OE-NC group (transfection of the OE-DNMT1 vector NC).

### Reverse transcription quantitative polymerase chain reaction (RT-qPCR)

Total RNA in tissues and cells was extracted by the TRIzol kit (Invitrogen). The purity of RNA was determined by a NanoDrop 2000 spectrophotometer (Thermo Fisher Scientific, MA, USA). Complementary DNA was amplified by a reverse transcription kit (Applied Biosystems, Darmstadt, Germany). The primers (Table 1) were from Sangon (Shanghai, China). Fluorescent quantitative PCR was carried out with the SYBR Premix Ex Taq^TM^II kit (Takara, Shiga, Japan). Fluorescence quantification was performed on a Bio-Rad CFX96 instrument (Bio-Rad, CA, USA). The PCR products were subjected to electrophoresis on a 2% agarose gel. Glyceraldehyde phosphate dehydrogenase (GAPDH) was used as the endogenous reference for HOTAIR, DNMT1, DNMT3A, DNMT3B, and PTEN. Data were analyzed by the 2-^ΔΔ^Ct method. The experiment was repeated three times.

**Table 1:** Primer sequence.

Gene	Sequence (5′→3′)
HOTAIR	Forward: GGTAGAAAAAGCAACCACGAAGC
	Reverse: ACATAAACCTCTGTCTGTGAGTGCC
DNMT1	Forward: AACCTTCACCTAGCCCCAG
	Reverse: CTCATCCGATTTGGCTCTTCA
DNMT3A	Forward: CCTGTGGGGAGCCTCAATGTTA
	Reverse: CTTGCAGTTTTGGCACATTCC
DNMT3B	Forward: CGGCTCTTCTTCGAATTTTACC
	Reverse: AGAACGGCCGGTCATCAC
PTEN	Forward: ATACCAGGACCAGAGGAAACC
	Reverse: TTGTCATTATCTGCACGCTC
GAPDH	Forward: TGAAGGTCGGAGTCAACGG
	Reverse: CTGGAAGATGGTGATGGGATT

*HOTAIR* HOX antisense intergenic RNA, *DNMT1* DNA methyltransferase 1, *DNMT3A* DNA methyltransferase 3A, *DNMT3B* DNA methyltransferase 3B, *PTEN* phosphatase and tensin homolog, *GAPDH* glyceraldehyde phosphate dehydrogenase, *18SrRNA* 18S ribosomal RNA.

### Western blot assay

Total protein was extracted, and the protein concentration was measured by the bicinchoninic acid method. A sodium dodecyl sulfate separating gel and spacer gel (10%) were prepared. The sample was mixed with loading buffer and boiled at 100 °C for 5 min. The protein was separated by electrophoresis and transferred to a nitrocellulose membrane; the membrane was sealed and incubated with primary antibodies against DNMT1 (1:1000, Abcam, MA, USA), PTEN (1:1000), and GAPDH (1:1000, Cell Signaling Technology, MA, USA) overnight. Then, the membrane was incubated with a secondary immunoglobulin G antibody (IgG, 1:2000, Boster, Hubei, China), labeled with horseradish peroxidase and observed postdevelopment. GAPDH was used as the loading control. The gray value of the band image was detected by the Quantity One software system. The ratio of the gray value of the target band to GAPDH was analyzed. The experiment was repeated three times.

### Methylation-specific polymerase chain reaction (MSP) assay

The MSP assay was performed with the DNeasy Tissue Kit according to the manufacturer’s instructions (Qiagen company, Hilden, Germany, No69504). Genomic DNA was extracted from cells and stored at −20 °C. The extracted DNA samples were in the range of 1.7–1.9. The DNA sample was treated with sulfite and purified by the CpGenome (tm) DNA Modification Kit (Chemicon International Inc., CA, USA). Methylated modified PTEN DNA was used as the template for PCR amplification with two pairs of primers (Invitrogen), PTEN methylation (forward: 5′-GGTTTCGGAGGTCGTCGGC-3′; reverse: 5′-CAACCGAATAATAACTACTACGACG-3′ [amplification length was 155 bp]) and non-methylation (forward: 5′-TGGGTTTTGGAGGTTGTTGGT-3′; reverse: 5′-ACTTAACTCTAAACCACAACCA-3′ [amplification length was 173 bp]). Water was used as the NC. The PCR products (5 μL) were subjected to electrophoresis on a 3% agarose gel and observed under an ultraviolet lamp. The experiment was repeated three times.

### RNA-binding protein immunoprecipitation (RIP)

Cells were lysed, and the same amounts of RIP lysate and the corresponding antibodies (DNMT1, 1:200, Abcam), EZH2 (1:100) and IgG (1:1000, both from Cell Signaling Technology) were loaded onto magnetic beads. Then, the bound RNA was purified and analyzed by RT-qPCR. The experiment was repeated three times.

### Chromatin immunoprecipitation (ChIP) assay

Cells transfected with sh-HOTAIR were cultured for 1 day, and then, ChIP assays of K562 and KCL-22 cells were carried out with EZ-Magna ChIP reagent (Millipore, MA, USA). A nonspecific IgG antibody (1:1500, Cell Signaling Technology) was employed as the NC. PTEN gene fragments were quantified by qPCR. Hot-Start Taq polymerase (Takara) was used in a 20 μL PCR reaction. The PCR products (10 μL) and a 100-bp DNA maker were separated by 2% agarose electrophoresis for comparison of abundance. The experiment was repeated three times.

### Subcellular localization experiment

The subcellular localization of HOTAIR was predicted by a bioinformatics website (http://lncatlas.crg.eu/). K562/KCL-22 nuclei and cytoplasms were separated by the PARIS kit (Thermo Fisher Scientific). The relative expression of HOTAIR was verified by RT-qPCR. The experiment was repeated three times.

### Fluorescence in situ hybridization (FISH) assay

Slides (10 mm × 10 mm) were placed in 24-well plates. Well-grown K562 and KCL-22 cells were seeded at 6 × 10^3^ cells/well. FISH assays were performed with LncRNA FISH Probe Mix (Red) and its matching kit (RiboBio). The cells were suspended in a 4 °C paraformaldehyde solution, cleaned with 0.1% Triton X-100, prehybridized with prehybridization solution, hybridized with a HOTAIR probe, rinsed with 2× sodium citrate buffer, stained with the 4′-6-diamidino-2-phenylindole fluorescent dye, and finally, observed under a fluorescence microscope. The experiment was repeated three times.

### Cell counting kit (CCK)-8 assay

Cell viability was tested by the CCK-8 kit (Beyotime Institute of Biotechnology, Shanghai, China). Cells (5 × 10^3^ cells, 100 μL) at 80% confluence were cultured in a 96-well plate and then incubated for 3 h in CCK-8 reagent after culturing for 0, 12, 24, 36, and 48 h. Optical density (Multiscan FC, Thermo Fisher Scientific) was detected at 450 nm. The experiment was repeated three times.

### Colony formation assay

Cells were trypsinized, and single cells were resuspended. Cells (4000 cells/group) were cultured for 14 days in a 60-mm culture dish with 10% FBS. Colonies were fixed, dyed with 0.5% crystal violet staining solution, imaged, and counted under a microscope. The experiment was repeated three times.

### Transwell assay

Diluted Matrigel (50 μL) was spread on the upper chamber of a Transwell chamber. Cells were suspended in serum-free medium, and a cell suspension (200 μL, 2 × 10^5^ cells/mL) was added to the upper chamber (600 μL cell medium with 10% FBS was added to the lower chamber). After culturing for 48 h, cells were fixed with absolute methanol and dyed with 0.1% crystal violet staining solution. Six fields of view were randomly selected under an inverted microscope to count the number of cells that crossed the membrane. The same method was used to detect cell migration, except that the upper chamber was not coated with Matrigel. The experiment was repeated three times.

### Flow cytometry

Cell cycle: Cells were prepared as single-cell suspensions, seeded in a 6-well plate at 1 × 10^5^ cells/well and fastened with 70% cold ethanol overnight. All steps were performed according to the instructions of the cell cycle detection kit (Dojindo Laboratories, Kumamoto, Japan). The propidium iodide (PI) staining method was adopted with a BD FAC Calibur flow cytometer to detect the cell cycle.

Cell apoptosis: Cells were trypsinized, centrifuged and resuspended. Cells were suspended in 100 μL 1× binding buffer, mixed with 5 μL annexin V-fluorescein isothiocyanate and 5 μL PI staining solution for 10 min, mixed with 400 μL 1× binding buffer and detected by a flow cytometer. The experiment was repeated three times.

### Tumor xenograft in nude mice

A total of 30 female BALB/c-nu/nu nude mice aged 4–5 weeks weighing 16–18 g were obtained from the Animal Research Center of the Chinese Academy of Sciences (Shanghai, China). Mice were kept at a constant temperature of 18–22 °C with a constant humidity of 50–80% and were provided free access to food and water. Mice were distributed into ten groups (*n* = 3). One day before the experiment, nude mice were exposed to a SliPrecise linear accelerator (Elekta Instruments AB, Stockholm, Sweden). The total dose was 350 cGy per nude mouse to increase the rate of successful cell transplantation. At a cell density of 5 × 10^7^ cells/mL, the cell suspension (1 × 10^7^ cells/200 μL) was injected subcutaneously into the axillary back of each nude mouse. After 3–5 days, tumor growth was observed. Nude mice were weighed, and tumor volume was measured every 4 days. Nude mice were euthanized 20 days after injection.

### Statistical analysis

All data were explicated by SPSS 21.0 software (IBM Corp. Armonk, NY, USA). The measurement data were conveyed by mean ± standard deviation. The data distribution was tested for normality. Comparison between two groups was conducted by independent sample *t*-test, and comparison among multiple groups was conducted by one-way analysis of variance (ANOVA) followed by Tukey’s multiple comparisons test. The association of HOTAIR expression with clinicopathological features of CML was determined by chi-square test. *P* value < 0.05 was indicative of statistically significant difference.
